# Early analysis of surrogate endpoints for metastatic melanoma in immune checkpoint inhibitor trials

**DOI:** 10.1097/MD.0000000000003997

**Published:** 2016-07-01

**Authors:** Fausto Petrelli, Andrea Coinu, Mary Cabiddu, Karen Borgonovo, Mara Ghilardi, Veronica Lonati, Sandro Barni

**Affiliations:** Oncology Department, Oncology Division, ASST Bergamo Ovest, Treviglio (BG), Italy.

**Keywords:** advanced melanoma, immunotherapy, ipilimumab, overall survival, PD-L1, surrogate endpoints

## Abstract

Recent major phase III trials led to the approval of immune checkpoint inhibitors (ipilimumab, pembrolizumab, and nivolumab) in metastatic malignant melanoma (MM). We aim to assess whether median progression-free survival, and 1 and 2-year overall survival (OS) rates are reliable surrogate endpoints for median OS through a meta-analysis of published trials involving immunotherapy. A systematic literature search in PubMed, EMBASE, Web of Science, and SCOPUS of published phase II to III trials with immunotherapy as the treatment for MM was conducted. Adjusted weighted linear regression was used to calculate Pearson correlations (*R*) between surrogates and median OS, and between treatment effects on surrogates and median OS. A total of 13 studies involving 3373 patients with MM were identified. The correlation of progression-free survival with OS was not significant (*R* = 0.45, *P* = .11). Conversely, the correlation between 1-year OS and median OS was very strong (*R* = 0.93, 95% confidence interval [CI] 0.84–0.96, *P* < .00001), as was the correlation between 2-year OS and OS (*R* = 0.79, 95% CI 0.51–0.91, *P* = .0001). The correlation between the treatment effects on 1-year OS and OS was also significant (*R* = −0.86, 95% CI −0.3 to 0.97, *P* = .01). Similar results were obtained for 2-year OS. According to the available study data, 1-year OS rate could be regarded as a potential surrogate for median OS in novel immunotherapy trials of metastatic MM. Waiting for ongoing studies (e.g., pembrolizumab), we suggest that this intermediate endpoint could be considered as a potential primary endpoint in future clinical trials.

## 1 Introduction

Metastatic malignant melanoma (MM) is a disease with a poor prognosis. Traditionally, the backbone agent of treatment has been dacarbazine.^[1,2]^ Recently, systemic therapy with immune response checkpoint inhibitors (ipilimumab, nivolumab, and pembrolizumab) has increased 1 and 2-year survival compared with the standard treatment, particularly for the v-Raf murine sarcoma viral oncogene homolog B (BRAF)–wild-type subgroup. There are also some long-term survivors; in particular, a 20% of those treated with nivolumab and ipilimumab are alive at 3 to 5 years.^[3–7]^ In these recent trials, especially with nivolumab, median overall survival (OS) rates have not often been reached, due to the late control of the disease and a short median follow-up period (9–12 months), with some delayed responses linked to immune system re-activation.^[8,9]^ In a recently published randomized phase III study comparing nivolumab + ipilimumab versus ipilimumab alone, a 9-month increase in median progression-free survival (PFS) has still not provided enough events for an OS analysis.^[9]^

Although OS is the gold-standard measurement of treatment efficacy in most cancers, the evaluation of OS in clinical trials concerning metastatic MM requires a lengthy follow-up due to improvements in survival time with immune checkpoint inhibitors. Moreover, estimates of OS are increasingly impacted by treatment cross-over or maybe re-challenge with immunotherapy (e.g., ipilimumab) or use of Mitogen-activated protein kinase kinase (MEK) inhibitors.^[10,11]^ The median pooled OS with ipilimumab therapy is about 12 months, with a 50, 30, and 26% 1 to 2 and 3-year OS in treatment-naive patients.^[6]^ In a pooled analysis of 42 phase II trials published in a preimmune checkpoint inhibitor era, the combined median OS was 6 months, whereas 1-year OS was 25%.^[12]^

There has been a significant interest in validating surrogate endpoints for OS in advanced cancers, particularly in tumors with several lines of therapy available and a long natural history (e.g., breast cancer). Utilized as a substitute for another clinical endpoint, a surrogate is a measure that is expected to predict a sufficient clinical benefit in terms of outcome (OS). Correlation analyses have validated surrogate endpoints for OS in MM treated with dacarbazine as a control arm,^[13]^ but immunotherapies were included in only 1 trial. In this analysis of randomized, dacarbazine-controlled trials, a strong correlation between PFS and OS was found. If this would be confirmed in trials including modern therapies (immune checkpoint inhibitors), surrogacy could permit to design smaller randomized trials and could avoid withdrawal of potentially effective new treatments from future development. Typically evaluated as a secondary endpoint in oncology trials, PFS represents an attractive candidate for a surrogate endpoint, because it only measures the effect of the study drug and is not influenced by subsequent treatments (or cross-overs) that patients receive. Until today, the relationship between PFS and OS in MM treated with immunotherapy remains unclear. Furthermore, novel anticancer drugs have become available for MM, and the relationship between postprogression survival (PPS) and OS could dilute any survival benefit that is potentially observed with immunotherapy. For example, in breast cancer, where several lines of therapy are usually prescribed, OS benefit with first-line agents is difficult to capture in the presence of prolonged PPS.^[14]^ In this and other diseases where natural history of cancer is chronically prolonged (as recent data in MM have clearly shown), intermediate endpoints could reliably surrogate OS. However, responses to immunotherapy are sometimes late, with initial forms of pseudo-progression and subsequent shrinkage of metastases. Ipilimumab was actually associated with different patterns of responses, including a response after an increase in the total tumor burden, and also a response in the presence of new lesions, and these were all associated with a favorable outcome.^[15]^ As a consequence, different endpoints such as 1 and 2-year OS could be validated as surrogates.

The purpose of this study was to evaluate the relationship in MM between median PFS, 1 and 2-year OS rates, and median OS to validate them as surrogate endpoints for OS. A systematic review and arm-level analysis evaluated the association between median OS and 1 and 2-year OS rates, and median PFS in clinical trials of immune checkpoint inhibitors for MM.

## 2 Materials and methods

### 2.1 Literature search and study selection

A systematic literature search was conducted of PubMed, Web of Science, SCOPUS, and Embase up to July 3, 2015. Ethical approval was not required because it was not a human research study. The search terms included “melanoma” and CTLA-4 or PD-L1 or PD-1 “melanoma”[All Fields] and (“ctla-4 antigen” [MeSH Terms] or (“ctla-4” [All Fields] and “antigen” [All Fields]) or “ctla-4 antigen” [All Fields] or “ctla 4” [All Fields]) or (“antigens, cd274” [MeSH Terms] or (“antigens” [All Fields] and “cd274” [All Fields]) or “cd274 antigens” [All Fields] or (“pd” [All Fields] and “l1” [All Fields]) or “pd l1” [All Fields]) or PD-1 [All Fields]).

The search was limited to phase II to III clinical trials published in the English language. Two researchers (FP and AC) reviewed each abstract and text against the study inclusion and exclusion criteria. Any disagreement was resolved with the senior author (SB). Studies were included if they evaluated immune checkpoint inhibitors (anti-cytotoxic T-lymphocyte antigen 4 [CTLA-4] or anti-programmed death 1 receptor [PD-1]/programmed death ligand 1 [PD-L1] drugs) for only cutaneous MM, reported both median OS and either median PFS (or time to progression) or 1 and/or 2-year OS rates. Retrospective or prospective series and phase I studies were excluded. In the event that a study was published in multiple articles or abstracts, the most recent data were used.

### 2.2 Data extraction

Data were extracted for study design, year of publication, sample size per treatment arm, treatment, and treatment line for each included study. Data on the response rate, median OS, 1 and 2-year OS rates, and median PFS were also collected.

### 2.3 Statistical analysis

The primary clinical outcomes were PFS, and 1 and 2-year OS rates. Our analysis used OS and PFS (or time to progression) data as defined and reported in the selected trials. Data on the median PFS and the proportion of patients alive at 1 and 2 year per treatment arm were extracted from each trial as surrogate endpoints for the analysis. The hazard ratio (HR) of the PFS and the difference (delta) in 1 and 2-year OS between the experimental and the control arms were used as the treatment effect estimate of clinical outcome. Two correlations were calculated between the summary statistics to determine surrogacy according to methods previously reported^[16–18]^ Correlations were achieved using weighted linear regression with the log transformation of the HRs and weights proportional to the sample sizes. The first approach, termed outcome surrogacy, computed the correlation between median PFS, 1 and 2-year OS rates (%), the potential surrogate endpoints, and median OS. The correlation was evaluated over all the treatment arms and is described as *R* (Pearson correlation coefficient). A strong or very strong correlation (*R* > 0.6–0.8 or >0.8) would be consistent with surrogacy for OS.^[19]^ The R-squared (*R*^2^) determination coefficient (the proportion of variability in OS explained by the variability of the surrogate endpoint) was also presented. The second approach, termed trial-level surrogacy, assessed the correlation between the reported treatment effects on a surrogate (logHR for PFS and delta in 1 and 2-year OS%), and those on OS (logHR for OS), which is the relevant endpoint. A very positive correlation would support PFS and 1 or 2-year OS as surrogate measures for OS.^[19]^

Furthermore, to explore the impact of other study variables, additional analyses were performed that stratified treatment arms by treatment line (first-line only); treatment type (ipilimumab); and (iii) phase III trials alone. The 95% confidence intervals (CIs) were obtained using the percentile bootstrap. All the reported *P* values correspond to 2-sided tests, and those that were less than 0.05 were considered to be statistically significant. Analyses were performed with the NCSS 2007 software (version 07.1.21, released June 1, 2011).

## 3 Results

**Figure 1:**
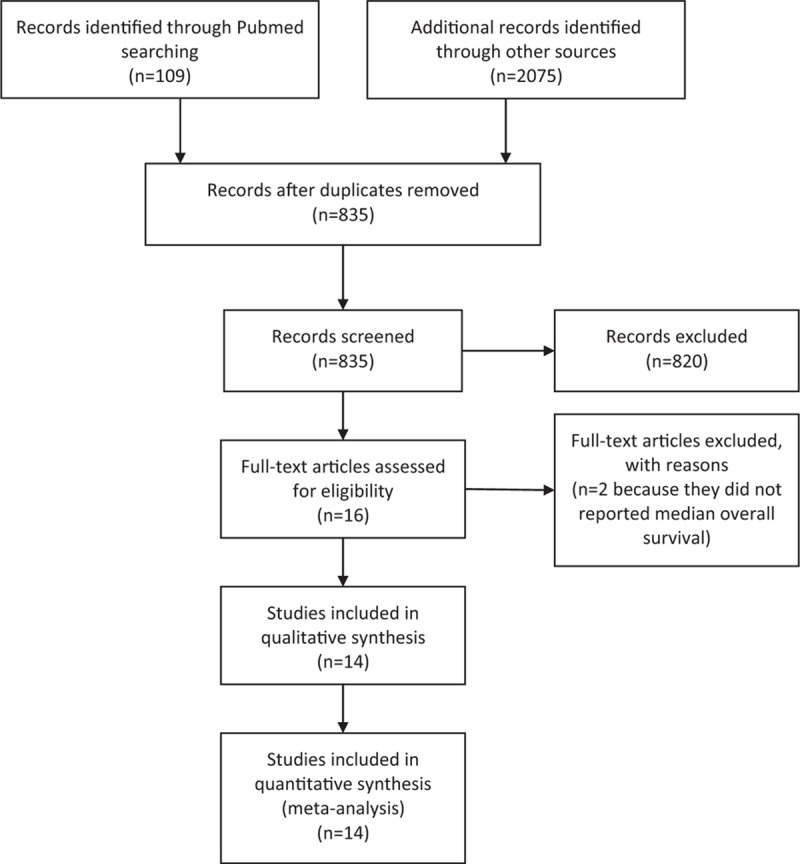
Flow diagram summarizing the strategy used to identify eligible studies.

As per the systematic literature review, a total of 2184 publications were analyzed (Fig. 1), with 13 studies considered for inclusion in the final analyses.^[3,4,20–32]^ Most of the studies were randomized phase II (n = 5) or III clinical trials (n = 4), although n = 4 single-arm phase II studies were included. The evaluated immune checkpoint inhibitors included ipilimumab alone or in combination (n = 10 trials), and nivolumab and tremelimumab in n = 1 and n = 2 trials, respectively. In the control arms of randomized trials, dacarbazine, a chemotherapy drug chosen at the discretion of the investigator (either dacarbazine or temozolomide), gp100, and ipilimumab alone were the agents of choice in n = 2, n = 1, n = 1, and n = 7 arms, respectively.

These studies involved 26 treatment arms and 3373 patients (Table 1). There were between 21 and 676 patients with advanced or metastatic MM across the study treatment arms, and the median reported OS rates ranged from 3.7 to 19.3 months. In n = 1 study, the median OS in experimental arm was not available, but the survival data of control arm were included in outcome surrogacy analysis. The median reported values for median PFS ranged from 1.2 to 5.1 months. The 1 and 2-year OS rates ranged from 19% to 72.9% and from 10% to 41.7%. Among these trials, the n = 7 trial involved pretreated patients, n = 4 treatment-naive patients, and n = 2 both untreated and previously treated patients. The pooled median response rate, PFS, OS, 1 and 2-year OS were 10%, 2.69 months, 11.2 months, 45.8%, and 24%, respectively. In the n = 1 study, time-to-progression instead of PFS was presented.

**Table 1:**
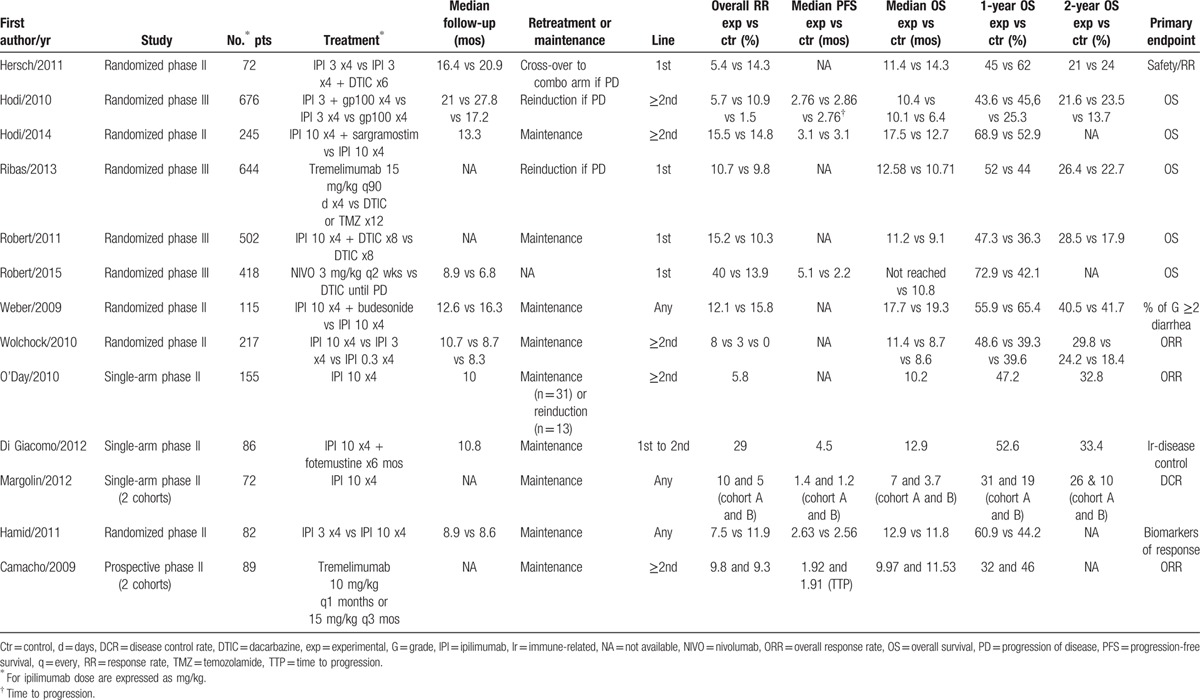
Characteristics of included studies.

The evaluation, as reported in the “Materials and methods” section, was performed using 2 different steps: the first considered the simple correlation between PFS, the 1 and 2-year OS%, and OS (reported as medians in months) for all treatment arms. The second step, which was necessary to demonstrate surrogacy, evaluated if any increase in PFS and the delta in 1 and 2-year OS% (the surrogate candidates) were fully captured by an increase in median OS (the main endpoint).

### 3.1 Outcome surrogacy

**Figure 2:**
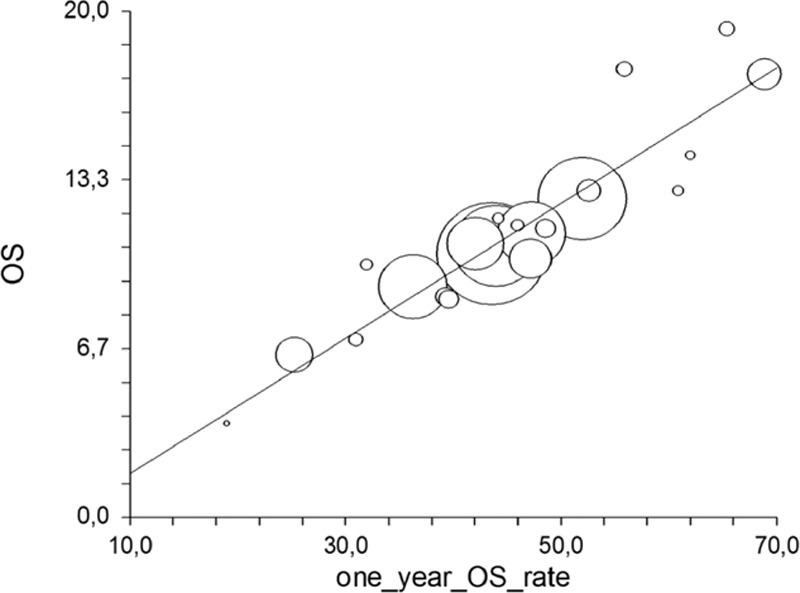
Correlation of 1-year with median overall survival.

**Figure 3:**
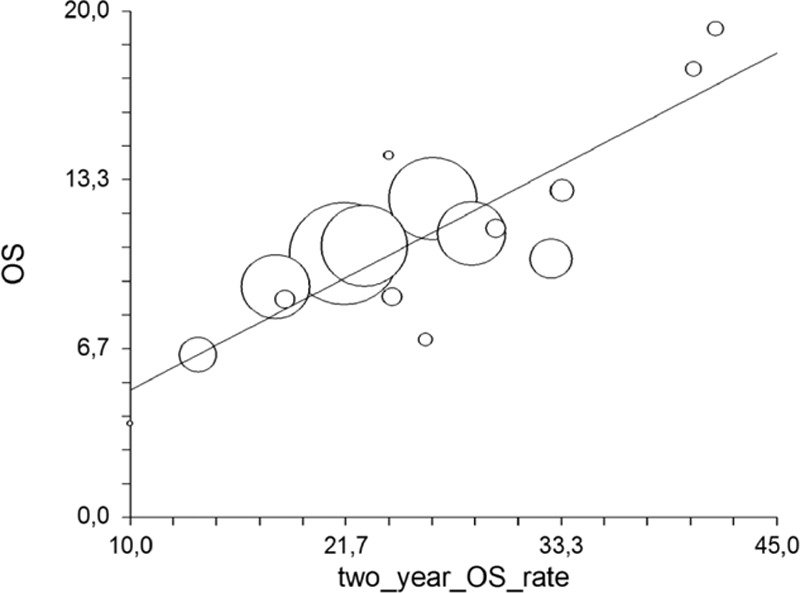
Correlation of 2-year with median overall survival.

Among a total of 28 arms available, the values for the PFS/OS correlation were reported in n = 13 trials. In the analysis of all the treatment regimens, the PFS correlated weakly with OS (*R* = 0.45, 95% CI −0.12 to 0.78, *P* = .11). The *R*^2^ values were 0.21 (*P* = not significant). The correlation between the 1-year OS%/OS was available for n = 25 arms and was very strong (*R* = 0.93, 95% CI 0.84–0.96, *P* < .00001; Fig. 2); *R*^2^ was 0.86 (*P* < 0.00001). The correlation between the 2-year OS%/OS was strong (n = 18 arms) (*R* = 0.79, 95% CI 0.51–0.91, *P* = .0001; Fig. 3); *R*^2^ was 0.63 (*P* = 0.0001).

Restricting the analysis to the ipilimumab-alone studies (n = 20 arms), the correlation of PFS with OS was poor (*R* = 0.51, *P* = not signifcant); instead, the correlations of the 1 and 2-year OS% with median OS were good (*R* = 0.93, *P* < 0.00001; and *R* = 0.81, *P* = 0.0001).

An analysis of the phase III trials and first-line studies was not performed because only 4 were available.

### 3.2 Trial-level surrogacy

**Figure 4:**
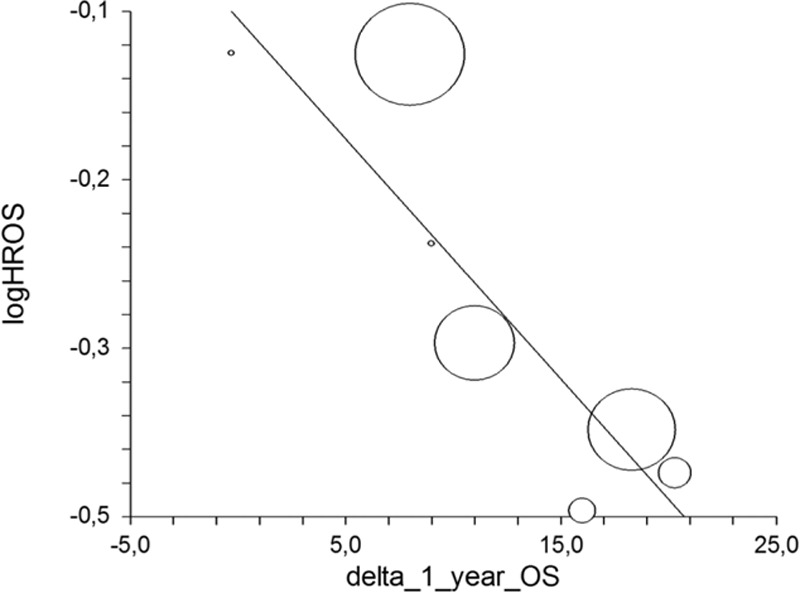
Treatment effect on delta 1-yeas overall survival (OS) with log hazard ratio (logHR) of median OS.

A total of 4 pairs of HRs for PFS and OS between the treatment arms were reported in n = 4 randomized trials, and so this correlation was not performed due to the paucity of data. The correlation deltas for the 1 and 2-year OS%/logHR OS were available for 7 and 6 pairs of comparisons. The correlation was very strong for the delta 1-year OS/logHR OS (*R* = −0.86, 95% CI −0.3 to 0.97, *P* = 0.01, *R*^2^ = 0.75; Fig. 4) and also for the delta 2-year OS/logHR OS correlation (*R* = −0.83, 95% CI −0.07 to 0.97, *P* = 0.03, *R*^2^ = 0.70).

The slope of the regression equation, namely the estimated change in the HR of OS per unit change in the rate of 1-year OS, was −0.019, with a standard error of 0.0667 (logHR OS = [−0.0587] + [−0.019]^∗^delta 1-year overall survival (YOS)). This means that a treatment associated with a 10% increase in 1-year OS translated into an approximate 19% reduction in the risk of death. Similarly, the slope of the regression equation, and the estimated change in the HR of OS per unit change in the 2-year OS rate, was −0.0364, with a standard error of 0.0911 (logHR OS = [−0.0167] + [−0.0364]∗delta 2-YOS). This means that a treatment associated with a 10% increase in 2-year OS translated into a 36.4% reduction in the risk of death.

For ipilimumab, only studies with a correlation delta of 1 and 2-year OS with logHR OS were different. The first (1-year OS) was strongly correlated (*R* = −0.92, 95% CI −0.43 to 0.98, *P* = .0076, *R*^2^ = 0.86), whereas the second was not correlated at all (*R* = −0.5, 95% CI 0.58–0.92, *P* = .38, *R*^2^ = 0.25).

## 4 Discussion

The use of surrogate endpoints in advanced cancers may facilitate the earlier analysis of trial data and provide more straightforward estimates of efficacy of any new drug tested in randomized, even if it does not eliminate the impact of cross-overs in the event of disease progression, in particular, with highly effective therapies. Recently, for example, the first-line trial comparing nivolumab to dacarbazine revealed a 30% difference for 1-year OS (72% vs 42%), with the anti-PD-1 antibody compared with chemotherapy, even if median OS was not achieved in the experimental arm at a median follow-up of 9 months. However, both European and US health authorities have approved nivolumab for the treatment of advanced unresectable or metastatic MM in adults, regardless of BRAF status, even in the absence of an actually demonstrable gain in median OS. The present analysis reveals that 1 and, to a lesser extent, 2-year OS rates are strong candidates for the role of surrogate endpoints for OS in trials treating metastatic MM with immunotherapy. Essentially, it is expected that fit and younger patients, with no rapidly progressing visceral disease/brain metastases and alive at 12 months, are those prognostically favored. Conversely, PFS is weakly correlated with OS.

Immunotherapy has revolutionized the treatment of advanced MM, which previously limited life to a few months. The therapy is associated with the prolonged stabilization of long-lasting disease, even after drug interruption, and there are some long-term survivors at 3 and 5 years.^[6]^ With older agents (e.g., dacarbazine), the responses were short-lived and the disease was associated with rapid progression and death, particularly in patients with a poor performance status, and brain and visceral metastases.^[12]^ In 2014, Flaherty et al^[13]^ found a robust correlation between PFS and OS in trials in which dacarbazine was the comparator arm. In this analysis, only the trial by Robert et al^[25]^, comparing ipilimumab + dacarbazine to dacarbazine alone, included immune checkpoint inhibitors. We now expand this analysis by considering only recently published phase II to III trials that involve immunotherapies (both anti-CTLA-4 and PD-1 inhibitors) and report survival data. We have found a very strong association between the 1-year OS rate and median OS, and a very weak correlation of PFS with median OS. In particular, the regression equation shows that a clinical trial comparing a new treatment that is able to increase 1 and 2-year OS by 10% is powered to detect a 19% and 36% reduction in the risk of death. This analysis primarily addresses ipilimumab trials, because only 1 study among those included compared nivolumab as an experimental arm to other treatments. In particular, the correlation is so robust that the rate and increase in the 1-year OS rate accounted for 86% and 75% of the variability in the median OS and HR for death, respectively.

In a disease where the natural history has ultimately changed, with long-lasting responders and some long-term survivors, there are several reasons to consider 1 and 2-year OS rates as optimal intermediate and surrogate endpoints instead of PFS (or time to progression) or the objective tumor response. First, OS at predefined time points is objective, simple to capture, and could overcome technical difficulties in evaluating the response rate and timing of disease progression on radiological imaging with immunotherapies. A reduction in the tumor burden is typically not immediate with ipilimumab, although it is faster with nivolumab. However, the criteria for defining the progression of disease are not equivocal, and classical World Health Organization (WHO) benchmarks are perhaps inadequate. Indeed, in the immune-related response criteria, the appearance of new lesions does not always define a progression of disease, and any new lesion is incorporated in the total tumor burden size.^[33]^ Finally, in new trials combining 2 immunotherapies (nivolumab + ipilimumab),^[34]^ a dramatic increase in efficacy has been observed, with median PFS reaching 12 months, leading to an increase in median OS that is hard to measure with just a few months of observation. In a similar companion trial that compared the combination with ipilimumab alone, the median duration of the response and PFS were not actually achieved.^[9]^ A surrogate endpoint in this setting could be useful, particularly for the rapid approval of these new drug combinations. For the reasons depicted above, in fact, evaluating 1-year OS as the primary endpoint of such randomized trials (with median OS unlikely to be reached with early follow-up) is more useful and time-sparing. Furthermore, radiographical difficulties in estimating progression data make such intermediate endpoint appealing for future design of clinical trials. On the contrary, in a disease where early death occurs rapidly, however, 1 or 2-year OS could be superimposable to median OS.

Our analysis has some obvious limitations. First, this is a literature-based analysis and only hypothesis-generating, meaning that confirmation with individual patient data is necessary. Furthermore, there were limited follow-ups for most of the trials presented. Second, the heterogeneity of the trials could have influenced the analysis, because small-scale phase II trials and larger phase III studies were combined, with both naive and pretreated patients included. A separate analysis (for line of therapy and type of study) is of limited power, because little data are available and so was not performed. The data on ipilimumab trials are, conversely, the main part of the included series, robust, and reflect data from the long-term analysis of published trials presented by Schadendorf et al^[6]^ in early 2015, with nearly similar outcomes for pretreated and naive patients at 3 years. Third, this analysis only refers to immunotherapy-based treatments, and the results cannot be automatically applied to anti-BRAF agents. Fourth, the analysis primarily covered trials with anti-CTLA-A drugs, with only 1 study including the novel anti-PD-1 agent nivolumab that is currently the standard treatment in first-line therapy. Fifth, for a large published phase III trial,^[34]^ and in particular for those with the anti-PD1 agent pembrolizumab,^[35,36]^ final OS analysis is not still available for inclusion in the present study, and a further update of this analysis could even reinforce the strength of surrogacy. In both studies, however, OS at 1 year is about 70%, which was even higher than those reported in the included studies. However, lack of these relevant studies makes the present analysis a little too preliminary. Finally, it is debatable which is the best primary endpoint in MM. Similarly, 5-year OS (milestone) instead of median OS could be an appealing endpoint in MM treated with immunotherapy. In fact, in ipilimumab trials, almost double number of patients are alive at 5 years, and this can be more clinically relevant of a little, albeit significant, improvement in median OS.

However, some strengths of the research must be underlined. This is the first systematic analysis of surrogate endpoints in MM after the chemotherapy era, and covered more than 3000 fit patients enrolled in clinical trials. In trials with immunotherapy, we found that 1 and 2-year OS rates are strong surrogates for OS, accounting for most of the entire outcome variation. We included all trials with ipilimumab alone or in combination, except 2 recent nivolumab/ipilimumab phase III studies and phase I trials. Second, it seems to be confuted that PFS can be regarded as a formal surrogate. Third, the very strong correlation between the 1-year OS rate and OS suggests that this endpoint could be further explored as a potential surrogate of OS in MM when new therapies are explored in the near future.

In conclusion, in a lethal disease where limited options were available for many years, drugs that target the immune system have now prolonged the natural history of MM to a chronic illness. Survival postprogression, with the exposure to several lines of modern therapies, accounts now for almost the entire survival, and so the need for a surrogate exists. In patients with metastatic disease, several options are now available (namely ipilimumab, nivolumab, pembrolizumab, cytotoxics, interleukin-2, anti-MEK, and anti-BRAF agents in BRAF mutated MM), and so long-term survivors are a realistic possibility. Even if OS probably still drives any investigation of new drugs in randomized trials in MM, the present preliminary analysis of 13 trials, with the limitations of an immature follow-up data and lack of inclusion of some relevant studies, suggests that the rate of 1-year OS could be an appropriate surrogate endpoint for OS in any line of treatment for advanced MM using immunotherapies. Additional intermediate endpoints in MM, including tumor or patient-related clinicopathological or radiological biomarkers used for other diseases, should also be explored in advanced MM.

## References

[1] Huncharek M Caubet JF McGarry R Single-agent DTIC versus combination chemotherapy with or without immunotherapy in metastatic melanoma: a meta-analysis of 3273 patients from 20 randomized trials. *Melanoma Res* 2001; 11:75–81. 11254118 10.1097/00008390-200102000-00009

[2] Ives NJ Stowe RL Lorigan P Chemotherapy compared with biochemotherapy for the treatment of metastatic melanoma: a meta-analysis of 18 trials involving 2,621 patients. *J Clin Oncol* 2007; 25:5426–5433. 18048825 10.1200/JCO.2007.12.0253

[3] Robert C Long GV Brady B Nivolumab in previously untreated melanoma without BRAF mutation. *N Engl J Med* 2015; 372:320–330. 25399552 10.1056/NEJMoa1412082

[4] Hodi FS O’Day SJ McDermott DF Improved survival with ipilimumab in patients with metastatic melanoma. *N Engl J Med* 2010; 363:711–723. 20525992 10.1056/NEJMoa1003466 PMC3549297

[5] Maio M Grob JJ Aamdal S Five-year survival rates for treatment-naive patients with advanced melanoma who received ipilimumab plus dacarbazine in a phase III trial. *J Clin Oncol* 2015; 33:1191–1196. 25713437 10.1200/JCO.2014.56.6018 PMC5795709

[6] Schadendorf D Hodi FS Robert C Pooled analysis of long-term survival data from phase II and phase III trials of ipilimumab in unresectable or metastatic melanoma. *J Clin Oncol* 2015; 33:1889–1894. 25667295 10.1200/JCO.2014.56.2736 PMC5089162

[7] Topalian SL Hodi FS Brahmer JR Safety, activity, and immune correlates of anti-PD-1 antibody in cancer. *N Engl J Med* 2012; 366:2443–2454. 22658127 10.1056/NEJMoa1200690 PMC3544539

[8] Robert C Long GV Brady B Nivolumab in previously untreated melanoma without BRAF mutation. *N Engl J Med* 2015; 372:320–330. 25399552 10.1056/NEJMoa1412082

[9] Postow MA Chesney J Pavlick AC Nivolumab and ipilimumab versus ipilimumab in untreated melanoma. *N Engl J Med* 2015; 372:2006–2017. 25891304 10.1056/NEJMoa1414428 PMC5744258

[10] Lebbé C Weber JS Maio M Survival follow-up and ipilimumab retreatment of patients with advanced melanoma who received ipilimumab in prior phase II studies. *Ann Oncol* 2014; 25:2277–2284. 25210016 10.1093/annonc/mdu441 PMC4990834

[11] Ascierto PA Simeone E Sileni VC Clinical experience with ipilimumab 3 mg/kg: real-world efficacy and safety data from an expanded access programme cohort. *J Transl Med* 2014; 12:116. 24885479 10.1186/1479-5876-12-116 PMC4030525

[12] Korn EL Liu PY Lee SJ Meta-analysis of phase II cooperative group trials in metastatic stage IV melanoma to determine progression-free and overall survival benchmarks for future phase II trials. *J Clin Oncol* 2008; 26:527–534. 18235113 10.1200/JCO.2007.12.7837

[13] Flaherty KT Hennig M Lee SJ Surrogate endpoints for overall survival in metastatic melanoma: a meta-analysis of randomised controlled trials. *Lancet Oncol* 2014; 15:297–304. 24485879 10.1016/S1470-2045(14)70007-5 PMC4443445

[14] Broglio KR Berry DA Detecting an overall survival benefit that is derived from progression-free survival. *J Natl Cancer Inst* 2009; 101:1642–1649. 19903805 10.1093/jnci/djp369 PMC4137232

[15] Wolchok JD Hoos A O’Day S Guidelines for the evaluation of immune therapy activity in solid tumors: immune-related response criteria. *Clin Cancer Res* 2009; 15:7412–7420. 19934295 10.1158/1078-0432.CCR-09-1624

[16] Tang PA Bentzen SM Chen EX Surrogate end points for median overall survival in metastatic colorectal cancer: literature based analysis from 39 randomized controlled trials of first-line chemotherapy. *J Clin Oncol* 2007; 25:4562–4568. 17876010 10.1200/JCO.2006.08.1935

[17] Buyse M Molenberghs G Burzykowski T The validation of surrogate endpoints in meta-analyses of randomized experiments. *Biostatistics* 2000; 1:49–67. 12933525 10.1093/biostatistics/1.1.49

[18] Alonso A Van der Elst W Molenberghs G On the relationship between the causal-inference and meta-analytic paradigms for the validation of surrogate endpoints. *Biometrics* 2015; 71:15–24. 25274284 10.1111/biom.12245

[19] Bland JM Altman DG Calculating correlation coefficients with repeated observations: part 2: correlation between subjects. *BMJ* 1995; 310:633. 7703752 10.1136/bmj.310.6980.633 PMC2549010

[20] Hodi FS Lee S McDermott DF Ipilimumab plus sargramostim vs ipilimumab alone for treatment of metastatic melanoma: a randomized clinical trial. *JAMA* 2014; 312:1744–1753. 25369488 10.1001/jama.2014.13943 PMC4336189

[21] Ribas A Kefford R Marshall MA Phase III randomized clinical trial comparing tremelimumab with standard-of-care chemotherapy in patients with advanced melanoma. *J Clin Oncol* 2013; 31:616–622. 23295794 10.1200/JCO.2012.44.6112 PMC4878048

[22] Di Giacomo AM Ascierto PA Pilla L Ipilimumab and fotemustine in patients with advanced melanoma (NIBIT-M1): an open-label, single-arm phase 2 trial. *Lancet Oncol* 2012; 13:879–886. 22894884 10.1016/S1470-2045(12)70324-8

[23] Di Giacomo AM Ascierto PA Queirolo P Three-year follow-up of advanced melanoma patients who received ipilimumab plus fotemustine in the Italian Network for Tumor Biotherapy (NIBIT)-M1 phase II study. *Ann Oncol* 2015; 26:798–803. 25538176 10.1093/annonc/mdu577

[24] Hamid O Schmidt H Nissan A A prospective phase II trial exploring the association between tumor microenvironment biomarkers and clinical activity of ipilimumab in advanced melanoma. *J Transl Med* 2011; 9:204. 22123319 10.1186/1479-5876-9-204 PMC3239318

[25] Robert C Thomas L Bondarenko I Ipilimumab plus dacarbazine for previously untreated metastatic melanoma. *N Engl J Med* 2011; 364:2517–2526. 21639810 10.1056/NEJMoa1104621

[26] Hodi FS O’Day SJ McDermott DF Improved survival with ipilimumab in patients with metastatic melanoma. *N Engl J Med* 2010; 363:711–723.[Erratum in: N Engl J Med. 2010 Sep 23;363(13):1290]. 20525992 10.1056/NEJMoa1003466 PMC3549297

[27] O’Day SJ Maio M Chiarion-Sileni V Efficacy and safety of ipilimumab monotherapy in patients with pretreated advanced melanoma: a multicenter single-arm phase II study. *Ann Oncol* 2010; 21:1712–1717. 20147741 10.1093/annonc/mdq013

[28] Hersh EM O’Day SJ Powderly J A phase II multicenter study of ipilimumab with or without dacarbazine in chemotherapy-naïve patients with advanced melanoma. *Invest New Drugs* 2011; 29:489–498. 20082117 10.1007/s10637-009-9376-8

[29] Wolchok JD Neyns B Linette G Ipilimumab monotherapy in patients with pretreated advanced melanoma: a randomised, double-blind, multicentre, phase 2, dose-ranging study. *Lancet Oncol* 2010; 11:155–164. 20004617 10.1016/S1470-2045(09)70334-1

[30] Weber J Thompson JA Hamid O A randomized, double-blind, placebo-controlled, phase II study comparing the tolerability and efficacy of ipilimumab administered with or without prophylactic budesonide in patients with unresectable stage III or IV melanoma. *Clin Cancer Res* 2009; 15:5591–5598. 19671877 10.1158/1078-0432.CCR-09-1024

[31] Camacho LH Antonia S Sosman J Phase I/II trial of tremelimumab in patients with metastatic melanoma. *J Clin Oncol* 2009; 27:1075–1081. 19139427 10.1200/JCO.2008.19.2435

[32] Margolin K Ernstoff MS Hamid O Ipilimumab in patients with melanoma and brain metastases: an open-label, phase 2 trial. *Lancet Oncol* 2012; 13:459–465. 22456429 10.1016/S1470-2045(12)70090-6

[33] Wolchok JD Hoos A O’Day S Guidelines for the evaluation of immune therapy activity in solid tumors: immune-related response criteria. *Clin Cancer Res* 2009; 15:7412–7420. 19934295 10.1158/1078-0432.CCR-09-1624

[34] Larkin J Chiarion-Sileni V Gonzalez R Combined nivolumab and ipilimumab or monotherapy in untreated melanoma. *N Engl J Med* 2015; 373:23–34. 26027431 10.1056/NEJMoa1504030 PMC5698905

[35] Ribas A Puzanov I Dummer R Pembrolizumab versus investigator-choice chemotherapy for ipilimumab-refractory melanoma (KEYNOTE-002): a randomised, controlled, phase 2 trial. *Lancet Oncol* 2015; 16:908–918. 26115796 10.1016/S1470-2045(15)00083-2 PMC9004487

[36] Robert C Schachter J Long GV Pembrolizumab versus ipilimumab in advanced melanoma. *N Engl J Med* 2015; 372:2521–2532. 25891173 10.1056/NEJMoa1503093

